# A retrospective cohort study of bladder cancer following the COVID-19 pandemic: Are patients presenting with more aggressive disease?

**DOI:** 10.1016/j.amsu.2022.104430

**Published:** 2022-08-18

**Authors:** Steven Anderson, Kate Rigney, Leah Hayes, Paul Christopher Ryan, Vishwa Chaitanya, Prem Thomas Jacob, Mamoun Abdelrahman, Subhasis K. Giri

**Affiliations:** a Dept of Urology, University Hospital Limerick, Ireland; b University of Limerick, Ireland; c Royal College of Surgeons Ireland, Ireland

**Keywords:** Bladder cancer, Urothelial cancer, COVID-19, Public health, Urology, Uro-oncology

## Abstract

**Background:**

The COVID-19 pandemic has resulted in delays in the treatment of patients with urological malignancies. The management of bladder cancer (BC) in particular poses a significant challenge given the recurrent nature of the disease and the intense follow-up regime required for many cases. The aim of this study was to evaluate potential changes in the presentation and operative management of BC in our hospital following the pandemic.

**Materials and methods:**

This is a retrospective cohort study. Potential BC cases were identified through the histopathology database between March 2019 and February 2021. Details were obtained on patient demographics, procedure type such as biopsy, resection or excision, grade and stage of BC. Cases were divided into two groups: period one (pre-COVID between March 2019 and February 2020) and period two (post-COVID between March 2020 and February 2021).

**Results:**

A total of 207 procedures for confirmed BC were performed during the study period, 126 in period one and 81 in period two. New cases accounted for 52.4% (n = 66) and 53.1% (n = 43) of cases during periods one and two respectively. There was a higher rate of invasive disease (43.2% vs 26.2%) as well as high grade disease (47.4% vs 35.8%) in period two than in period one.

**Conclusion:**

Fewer BC procedures were performed in the COVID period. The higher rate of more advanced stage and grade of disease seen in period two suggests patients are presenting later. This should be considered when allocating resources in the management of non-COVID related diseases. Further studies are needed to assess the long-term impact of COVID-19 on bladder cancer outcome.

## Highlights

• Bladder cancer is a time-sensitive condition and delays in diagnosis are associated with worse outcomes.
• Fewer cases of bladder cancer were seen in the first year following the COVID-19 pandemic.
• There was no difference in waiting times from diagnosis to treatment in the year before and after the pandemic.
• In the year following COVID-19 patients presented with more aggressive bladder cancer than in the year before the pandemic.
• The more aggressive bladder cancer seen following COVID-19 is likely due to delays in patients presenting to primary care.

## Abbreviations

BC: Bladder cancer
HGBC: High grade bladder cancer
NMIBC: Non-muscle invasive bladder cancer
UHL: University Hospital Limerick

## 1 Introduction

The COVID-19 pandemic has created one of the biggest challenges in healthcare in recent history. The impact of the disease has extended far beyond the millions of patients who have been infected with it to date. The combination of the fear of contracting COVID-19 coupled with the necessary changes seen in many acute and elective hospital services have resulted in significant challenges for patients presenting with non-COVID related illness. Several studies have suggested that patients with non-COVID related emergencies are presenting later and with more advanced disease following the pandemic [1,2]. Furthermore, the closure of elective theatres and outpatient endoscopy suites has resulted in significant increases in waiting times for urgent elective procedures [3].

While these issues have the potential to affect the management of all patients, it is particularly apparent in patients with a new diagnosis or history of BC, given the recurrent nature of the disease and the invasiveness of most surveillance protocols.

Bladder cancer represents the eleventh most common cancer worldwide, with an age-standardised mortality rate of 3.2 and 0.9/100,000 person/years for men and women respectively [4]. Despite modern improvements in imaging modalities and biomarkers, endoluminal evaluation of bladder with cystoscopy remains essential in the diagnosis of BC. Furthermore, while the majority of patients present with non-muscle invasive bladder cancer (NMIBC), delays in diagnosis and treatment are associated with disease progression, which itself conveys a significant increase in morbidity and mortality [5].

As such, attempting to minimise the impact of COVID-19 on bladder cancer patients remains a significant challenge for urology departments around the world. The aim of this study was to evaluate potential changes in the presentation, management and outcomes of BC patients in our institution following the pandemic.

## 2 Patients and methods

### 2.1 Overview and study design

This is a retrospective observational study. Potential BC cases were identified through the histopathology department in University Hospital Limerick (UHL). The electronic records of all bladder specimens taken between the March 15, 2019 and the March 16, 2021 were reviewed, and all benign bladder biopsies were excluded. Flexible cystoscopies were not included in this study. A database of patient demographics and clinicopathological characteristics was created and a retrospective review was undertaken. Institutional ethical approval was granted by the local ethics committee and the study was registered prior to publication.

The aim of the study was to analyse differences in the presentation and management of BC prior to Covid-19 (period one – March 1, 2019 to March 15, 2020) and following the Covid-19 pandemic (period two – March 16, 2020 to March 1, 2021).

The primary outcome measure was stage and grade of disease at presentation. Secondary outcome measurements were differences in the number of cases of BC and the length of time from diagnosis to treatment between the two study periods.

Work was reported in line with STROCSS criteria and the study was registered with a Research Registry (identifying number researchregistry8026) [6].

### 2.2 Statistical analysis

Statistical analysis was performed using IBM SPSS software, versions 25 (SPSS Inc, Chicago, IL). Distributions were summarised using frequencies, means and ranges unless otherwise stated. The Independent-samples *t*-test and Pearson's Chi-square test were used to assess the association between continuous and categorical variables respectively. A p-value less than 0.05 was considered statistically significant.

## 3 Results

### 3.1 Patient characteristics

A total of 207 endoscopic and open procedures for confirmed bladder cancer were performed during the study period, 126 in period one, and 81 in period two. The mean age at time of treatment was 71 years (SD 10.8, range 36–93), with no difference seen between the two time periods (p = 0.86). The mean number of cases per month reduced from 9.54 in period one to 6.67 in period two (p = 0.019). Endoscopic procedures, either transurethral resection of bladder tumours (TURBT) or rigid cystoscopy and cold-cup biopsies, accounted for 97.5% of cases (n = 202). The remaining cases were radical cystectomies and ileal conduit formations in patients with either high grade bladder cancer or muscle invasive disease (three during period one, and two during period two). There was no difference in the proportion of cases that were due to bladder cancer recurrences between period one and period two (47.6% vs 46.9% respectively, p = 0.65).

### 3.2 Tumour characteristics

Urothelial cell cancer was found in 97.5% of cases (n = 202). There were two cases of adenocarcinoma, two cases of small cell cancer, and one case of bladder B-cell lymphoma. There were no cases of squamous cell cancer in our series.

The majority of patients had early-stage disease, with 65.2% (n = 135) presenting with pTa disease, and 24.2% (n = 50) with pT1 disease. There was a significantly higher rate of more advanced disease seen following the COVID-19 pandemic. In period one, only 26.2% (n = 33) of patients had invasive disease (≥pT1) at the time of treatment, compared to 43.2% (n = 35) in period two (p = 0.01).

Similar findings were seen with respect to tumour grade. Most urothelial cancer cases were either low grade (56.7%, n = 114) or a papillary urothelial neoplasm of low malignant potential (1.5%, n = 3). There was a higher rate of high-grade bladder cancer (HGBC) following the pandemic. HGBC accounted for 35.8% (n = 44) of urothelial cancer cases in period one compared to 47.4% (n = 37) in period two (p = 0.01). The stage and grade of urothelial cancer at time of treatment are summarised in Table 1.

**Table 1:** Stage and grade of urothelial cancer at diagnosis.

	Period One *n (%)*	Period Two *n (%)*	Total *n (%)*
Stage			
pTis	3 (2.4)	1 (1.2)	4 (1.9)
pTa	90 (71.4)	45 (55.6)	135 (65.2)
pT1	23 (18.3)	27 (33.3)	50 (24.2)
pT2	10 (7.9)	8 (9.9)	18 (8.7)
Grade			
PUNLMP	1 (0.8)	2 (2.6)	3 (1.5)
Low Grade	78 (63.4)	36 (46.2)	114 (56.7)
High Grade	44 (35.8)	37 (47.4)	81 (40.3)
Unknown	1 (0.8)	3 (3.8)	4 (2.0)

### 3.3 Waiting times

The overall mean waiting times, calculated from the date of booking to the date of procedure, were comparable between period one (63 days) and period two (76 days) with no significant difference seen (p = 0.28). Similarly, no significant differences were seen in the waiting times for TURBT in new patients or those with recurrences during either time period.

## 4 Discussion

In this study there was a statistically significant higher rate of more advanced and more aggressive bladder cancer in the first year following the pandemic than in the year before it. The authors hypothesise that this finding is likely due to patients presenting later to both primary and secondary care. Over two years on from the first reported case of COVID-19, the impact of the pandemic continues to be felt around the world. For several months following the pandemic, patients found themselves repeatedly being told to remain at home unless severely unwell. This necessary and widely issued public health precaution has unfortunately resulted in delayed presentations of several non-COVID related illnesses, including many malignancies [1,2,7]. As such, many publications issued recommendations on how services should prioritise the management of different malignancies during the pandemic in order to minimise the impact on patient outcomes [8,9]. However, the efficacy of these protocols remains unclear, and there is a paucity of data within the literature on the impact of COVID-19 on many malignancies such as bladder cancer.

In this study there was a significant reduction in the number of BC surgeries performed in the first year following the pandemic. Although previously reported data from Ireland has documented a gradual decrease in the incidence of BC over the last 20 years, the reported decline of around 1% per year does not explain the significant decrease in BC seen in this series [10]. A possible explanation for this finding is the prolonged period of reduced elective activity seen in many hospitals following the pandemic. However, waiting times for those who underwent BC surgery did not significantly increase between the two time periods, which suggests that fewer patients are presenting with BC following COVID-19.

A concerning finding of this study is the higher rate of invasive disease and HGBC seen in both new patients and those with recurrences in period two. This contrasts somewhat with data published by Oderda et al. They retrospectively reviewed 767 patients who underwent TURBT between 2019 and 2020 and reported no differences in stage or grade of disease between the two years. However, it is important to note that BC patients in their study had more aggressive disease at baseline than in this present study, with 50% having invasive disease and 72% having high grade disease. Furthermore, although no differences in stage or grade of disease were seen for patients undergoing TURBT, they did report a statistically significant higher rate of node positive and non-organ confined disease in radical cystectomy patients following the pandemic compared to the year before. This further supports the findings of our study, in that patients undergoing treatment for BC have worse pathological features following the pandemic [11].

Elective surgical activity was significantly impacted by COVID-19 in our institution, as it was worldwide. Several protocols have been implemented across different institutions to ensure urgent care was still available to non-COVID patients. In the initial months following COVID-19 in our institution, urgent cancer cases were outsourced to a local private hospital, where the rate of COVID positive patients remained low. These outsourced cases were included in our database. As community numbers began to fall, elective surgery resumed in our institution for urgent and time-critical cases. All BC patients awaiting treatment were triaged as urgent and time-critical in our institution. As a result, the waiting times for treatment were not significantly different during the two time periods in this study. Waiting times were calculated, from the available data, as the time from presentation to treatment, as the time from symptom onset to presentation was not known. The authors believe that it is therefore the potential delays in presentation that explains the higher rate of invasive disease and HGBC seen following the pandemic in this study. This theory is supported by a recent study by Culpan et al. who analysed 407 patients undergoing cystoscopic surveillance for non-muscle-invasive bladder cancer (NMIBC) following COVID-19. They reported that a delay in cystoscopy of more than three months increased the probability of progression by 6.7-fold [12]. This is an important consideration for future resource allocation, as such delays can be expected globally. Spencer-Bowdage et al. reported that 49% of the 156 BC patients who responded to their survey, described a disruption to their treatment during their study period [13].

There are several limitations to this study. As a retrospective observational study there is an inherent risk of bias. It is possible that the reduced number of BC surgeries performed was due to factors other than COVID-19. BC data from our institution over the preceding years has not been published, however, extrapolating from national data, the decrease in cases seen over our study period far exceeds what could be reasonably explained by annual variations. Similarly, although the authors believe that delays in presentation are the most likely explanation for the differences in stage and grade seen between the two time periods, as the time from symptom onset to presentation is unknown, this cannot be confirmed. Finally, details on radiological staging were not reported as no patients diagnosed with NMIBC following treatment were found to have radiological evidence of locally advanced (≥T3 disease) or metastatic disease during our study period.

## 5 Conclusion

This study is one of the first to report on BC outcomes following COVID-19, and the first to do so in Ireland. There were fewer BC surgeries performed in the first year following the pandemic, with a higher proportion of patients diagnosed with invasive disease and HGBC than in the year prior to COVID-19. Although survival outcomes were not reported, stage and grade of disease are important prognostic makers for BC. The authors therefore caution against delaying the investigation, treatment and surveillance of BC patients during any potential future COVID-19 waves or other global health crises.

## Disclosures

The authors declare no conflicts of interest.

## Provenance and peer review

Not commissioned, externally peer-reviewed.

## Ethical approval

This study was granted ethical approval by the research and ethics comity of University Hospital Limerick.

Rec Ref: 084/2021.

## Sources of funding for your research

This study received no funding

## Registration of research studies

Name of the registry: Researchregistry.com
Unique Identifying number or registration ID: researchregistry8026
Hyperlink to your specific registration (must be publicly accessible and will be checked): https://www.researchregistry.com/browse-the-registry#home/. Click or tap if you trust this link.">https://www.researchregistry.com/browse-the-registry#home/

## Consent

As this was an observational retrospective review of anonymised patient data, informed consent was deemed not necessary by the local ethics comity.

## Guarantor

Steven Anderson.

## Declaration of competing interest

The authors disclose no conflicts of interest.
